# Social impact bonds: opportunities for funding health promotion and disease prevention

**DOI:** 10.1186/s12889-026-26916-1

**Published:** 2026-03-16

**Authors:** Stefánia Plankó, Emily Hulse, Maureen Rutten-van Mölken, Lucas Goossens, Sarah Wordsworth, Rositsa Koleva-Kolarova, Apostolos Tsiachristas, Stephen Wright, Júlia Zemplényiné Bartha, Balázs Nagy, Balázs Babarczy

**Affiliations:** 1 Syreon Research Institute, Budapest, Hungary; 2 Government Outcomes Lab, Blavatnik School of Government, University of Oxford, Oxford, UK; 3 Nuffield Department of Primary Care Health Sciences, University of Oxford, Oxford, UK; 4 Department of Health Technology Assessment, Erasmus School of Health Policy and Management, Erasmus University Rotterdam, Rotterdam, The Netherlands; 5 Health Economics Research Centre, Nuffield Department of Population Health, University of Oxford, Oxford, UK; 6 National Institute for Health Research (NIHR) , Oxford Biomedical Research Centre, Oxford, UK; 7 Department of Psychiatry, University of Oxford, Oxford, UK; 8 The Bartlett School of Sustainable Construction, University College London, London, UK; 9 Health ClusterNet, Amsterdam, The Netherlands; 10 Center for Health Technology Assessment, Center for Pharmacology and Drug Research & Development, Semmelweis University, Budapest, Hungary

**Keywords:** Health financing, Social impact bonds, Outcome-based contracting, Investment in health, Prevention, Health promotion, Disease prevention

## Abstract

**Context:**

Fiscal constraints, demographic shifts, and the growing burden of noncommunicable diseases (NCDs) challenge the sustainability of health systems. Innovative financing instruments, such as social impact bonds (SIBs), have been proposed to support prevention and health promotion, yet empirical evidence on their effectiveness remains limited.

**Objectives:**

To explore the advantages and disadvantages of SIBs for financing health prevention and promotion in OECD countries, drawing on perspectives from key stakeholders and evidence from the scientific and grey literature.

**Design:**

We conducted a qualitative exploratory study combining 15 semi-structured interviews with academics, consultants, investors, and representatives of public institutions, and a targeted review of 21 publications identified through a PubMed search and supplementary grey literature sources. Thematic analysis was applied to interview transcripts using a mixed inductive–deductive coding approach, and findings were triangulated with literature evidence.

**Results:**

SIBs offer flexibility in service delivery, foster performance monitoring, and may provide longer-term financial stability to service providers. They support the piloting of innovative interventions and enhance accountability in program implementation. However, expected cost savings for governments were rarely realized in practice. Concerns have also been raised, mainly in the theoretical literature, about rigid outcome targets, limited empirical evidence of effectiveness in NCD prevention, high transaction costs, the “wrong pocket” problem, and political vulnerability. In practice, while SIBs are considered more suitable for narrowly defined interventions with measurable intermediate outcomes than for population-wide prevention programs, their scale-up is possible, for example though outcome funds—pooling multiple SIB projects—were viewed as a potential solution for attracting larger investors and creating broader impact.

**Conclusions:**

SIBs offer a promising but contested approach to financing prevention and health promotion. Their success depends on careful intervention selection, transparent design, and adaptation to local institutional and political contexts.

**Clinical trial number:**

Not applicable.

**Supplementary Information:**

The online version contains supplementary material available at 10.1186/s12889-026-26916-1.

## Introduction

Recent rises in long-term real interest rates have escalated fiscal burdens on governments and strained the sustainability of public expenditures [1, 2]. Health care systems, already under persistent pressure, face further constraints, exacerbated by demographic transitions (population ageing, shrinking working-age cohorts) [3], demand for cutting-edge medical technologies [4], and growing burdens of noncommunicable diseases (NCDs).

NCDs, such as obesity, cardiovascular disease, diabetes, and cancer, are major drivers of morbidity, mortality, and health care costs globally, and tend to worsen disparities in health outcomes across socioeconomically stratified populations [5–7]. Addressing NCDs effectively requires sustained investment in prevention, early detection, and chronic disease management. Prevention efforts are especially important as they can reduce the cost of treatment while promoting sustainable economic growth. Based on analyses of OECD countries, an optimal preventive health investment of approximately 1.175% of GDP is recommended [8]. Yet, despite a temporary increase in preventive spending during the COVID-19 pandemic when healthcare expenditure on preventive care reached its peak at 0.67% of GDP in EU-27 in 2021, mainly due to vaccination programs [9], preventive programs currently represent only about 3% of total health expenditure in the European Union [4]. The most recent data available from 2023 indicates that healthcare expenditure on preventive care as a percentage of GDP in the EU-27 stands at 0.37% [9].

Given these constraints, many health systems are searching for financing and incentive mechanisms that maximize outcomes for public value. The OECD explicitly advocates the innovative use of external resources and the inclusion of public–private partnerships to improve efficiency [10]. Rooted in the contract-based approach of New Public Management [11–13], outcome-based payment models are one such strategy. Outcome-based payment is a general term covering any payment mechanism where financial compensation is connected, either partially or fully, to the achievement of specific outcomes. One option applies the mechanism of public-private partnerships (PPP), usually associated with capital-intensive investment in infrastructure, but extends it to the provision of service-intensive activities, including complex and often innovative social services. These partnerships link provider remuneration to outcomes achieved, thus intended to incentivize high-quality, cost-efficient care [3].

A specific form of external resource involvement in health or health-related prevention services is the use of Social Impact Bonds (SIBs), more recently also known as Social Outcome Partnerships in the UK, Contrats à impact in France, Pay for Success in the USA, and Social Benefit Bonds in Australia [14]. Social Impact Bonds can be defined similar to an outcome-based payment model with a third-party private investment paying for the upfront set-up costs of a service, with a government outcome funder repaying a full or partial return only if the prespecified outcomes are achieved. SIBs constitute a potential, though still underexplored, outcome-based payment model supported by external private sector funding. At present, SIBs account for less than 1% of wider outcome-based payments [15], but they are applied in an increasing variety of social policy settings. In a SIB, a commissioner, typically a public body, contracts one or more service providers to deliver evidence-based interventions. Service providers receive upfront financing by private social investors, with which they can provide the services for a pre-defined time period. At the end of the period, investors receive reimbursement and potential returns from the commissioner of the scheme, but only if pre-established, measurable outcomes are achieved as a result of the intervention [16, 17]. Applying the mechanism of incentives [18] to influence the behaviour of private contractors, this set-up is supposed to transfer risk from public payers to investors, and align the interests of all parties – commissioners, investors and service providers – in the achievement of pre-defined social outcomes.

Although SIBs have been tested in domains such as homelessness, employment, criminal justice, and education [19–21], they face serious criticism as to their (i) accountability, e.g., excessive bargaining power of private investors, and their ability to influence outcome measurement; (ii) measurement, e.g., difficulties of quantification, and metrics focusing the attention of service providers exclusively on specific aspects of the service; and (iii) cost-effectiveness, e.g., cost of capital and transaction costs [13].

Published evidence on the use of SIBs remains sparse, and most literature remains conceptual. This study aims at investigating the advantages and limitations of SIBs from the perspective of health promotion and disease prevention. Using primary qualitative data gathered via interviews, we explore real-world experiences and obstacles. We complement these findings with a targeted review of both academic and gray literature. This research has been conducted within the framework of the Horizon Europe–funded Invest4Health project, which aims to design and pilot alternative business models called Smart Capacitating Investment (SCI), for scaling prevention and health promotion initiatives.

By combining stakeholder insights and evidence synthesis, the study aims to explore whether and how SIBs could serve as viable financing tools in public health systems under fiscal constraint, especially for addressing the growing burden of NCDs.

## Methods

### Study design

We conducted a qualitative exploratory study triangulating primary data from expert interviews with secondary evidence from a targeted review of scientific and grey literature. This approach allowed us to examine the feasibility and implications of social impact bonds (SIBs) in health promotion and disease prevention in OECD countries. While the literature on SIBs is growing, it is dominated by conceptual analyses, with limited empirical research on their application in public health. Expert interviews provided practice-based perspectives from multiple stakeholder groups, while the literature review situated these perspectives within the broader evidence base.

### Expert interviews

Semi-structured interviews were conducted between October and December 2023 with selected experts using maximum variation sampling [22]. Sampling criteria included being either a representative of a philanthropic or social impact investor, or of a national or international policymaker, or being a researcher with expertise in novel methods of health financing and/or outcome-based financing. Potentially applicable institutions were listed through targeted literature review including, but not restricted to the University of Oxford’s Government Outcomes Lab (GOLab) website. Reasons for this source of sampling was GOLab’s expertise in outcome-based contracting and convening power hosting the Social Outcome Conference every year. Furthermore, interviewees were also identified through professional networks, targeted internet searches, and snowball recommendations. All interviews were conducted online via Microsoft Teams, lasted approximately one hour, and followed a pre-defined interview guide (Appendix B). With written informed consent (Appendix A), interviews were recorded and transcribed using Buzz offline audio transcription [23].

### Data analysis

Transcripts were analyzed using thematic analysis with a mixed inductive–deductive approach, with emerging codes later used deductively [24, 25]. Initially, three researchers independently coded a subset of three transcripts in Microsoft Word to generate data-driven individual codebooks (step 1).

Subsequently, a common codebook was created through consensus discussions. This common coding framework (Appendix C), containing themes derived from the consensus of initial coding, was applied to the full dataset using Microsoft Excel (step 2). Themes were developed so as to allow a comprehensive yet concise description of outcome-based investment models from a practical point of view. Criteria for theme development was a common phrase or important concept related to our research questions. We collated separate but similar codes into meaningful patterns across the dataset.

Finally, the content coded in step 2 was consolidated into a list of statements per theme in Microsoft Power Point (step 3). Researchers were cognizant of their world view and epistemological position, shaped by the majority being health economists by training and working for a market-based research company. However, diversity of views was also present throughout the research process, contributing to collective reflexivity. As health economists we had an initial understanding of how investment models in health promotion and disease prevention would work, yet we challenged our initial assumptions by allowing the interviewees data to inform new codes.

### Literature review

A rapid review with qualitative synthesis was conducted in MEDLINE (PubMed) on February 16, 2024. The search strategy employed Medical Subject Headings (MeSH) terms to ensure precision and comprehensiveness. Terms were derived from Peter Ramsden’s 2016 OECD working paper on outcomes-based financing [26] as well as themes emerging from the interviews. The search strategy focused on two categories:

1. Public health domain: using “public health” as a MeSH term.
2. Outcomes-based financing domain: terms included “social impact bond,” “health impact bond,” “outcome-based financing,” and “results-based contracts.”

The complete search string is provided in Appendix D.

To complement the PubMed search, we conducted backward citation tracking (snowballing) of relevant articles and incorporated grey literature sources as recommended by Wohlin et al. [27]. Grey literature included reports from international organizations (e.g., OECD, World Bank), evaluation studies, and policy documents. Additional materials were identified through targeted Google searches and recommendations from interviewees.

Sources were screened using four exclusion criteria at the title and abstract stage:

1. The article was unrelated to SIBs or outcome-based financing schemes.
2. The article did not focus on public health, health promotion, or disease prevention.
3. The article did not concern OECD countries.
4. The article did not provide an empirical or theoretical assessment of the financing instrument.

Articles meeting all inclusion criteria were subjected to full-text review, and data were extracted into Excel. Both peer-reviewed and grey literature were included to capture the breadth of empirical and conceptual insights available.

### Triangulation

Findings from interviews and literature were triangulated to ensure methodological rigor and provide a nuanced perspective on the use of SIBs in prevention and health promotion. In other words, every interview code was compared to the literature’s evidence. This allowed us to cross-validate themes and highlight convergences and divergences across stakeholders, investment models, and implementation contexts.

We summarized the advantages and disadvantages of SIBs, as emerging from the data, on three levels: effects on project outcomes, financial implications and system-level consequences. A Large Language Model (ChatGPT 5.0) was used for language and editing purposes during the preparation of the manuscript.

## Results

### General characterization of the results

In total, we analysed 15 interviews with academics, consultants, investors and representatives of public institutions. Their professional and geographic location is shown in Table 1. Saturation in terms of themes (i.e., topics and criteria through which investment models such as SIBs can be analysed) was reached at the first consensus meeting after the initial coding process of three transcripts. According to the partly deductive approach of the study, these were then applied through the rest of the research process; no need for new themes emerged from the data. Inter-coder reliability was ensured via continuous meetings and discussions within the research group.

**Table 1:** Summary information of the interviewees

Interviewee ID	Profession	Geographical location
1	University researcher	United Kingdom
2	University researcher	France
3	University researcher	United Kingdom
4	University researcher/consultant	Spain
5	Independent Researcher	United Kingdom
6	Investor - Social banking	Austria
7	Investor - Impact investing	Belgium
8	Representative of a public body	European Union -wide
9	Representative of a public body	Portugal
	Consultant	Italy
11	Consultant	Canada and Global
12	Investor	Norway
13	Investor – Social banking	Hungary
14	Representative of a public body	European Union -wide
15	Representative of a public body	European Union -wide

The themes derived from initial, free coding categories were the following:

- Stakeholders.
- Investment incentives and business models.
- Timeframes, investment size and financial returns.
- Impact measurement and scale-up.
- Advantages, disadvantages, and risks.

**Fig. 1:**
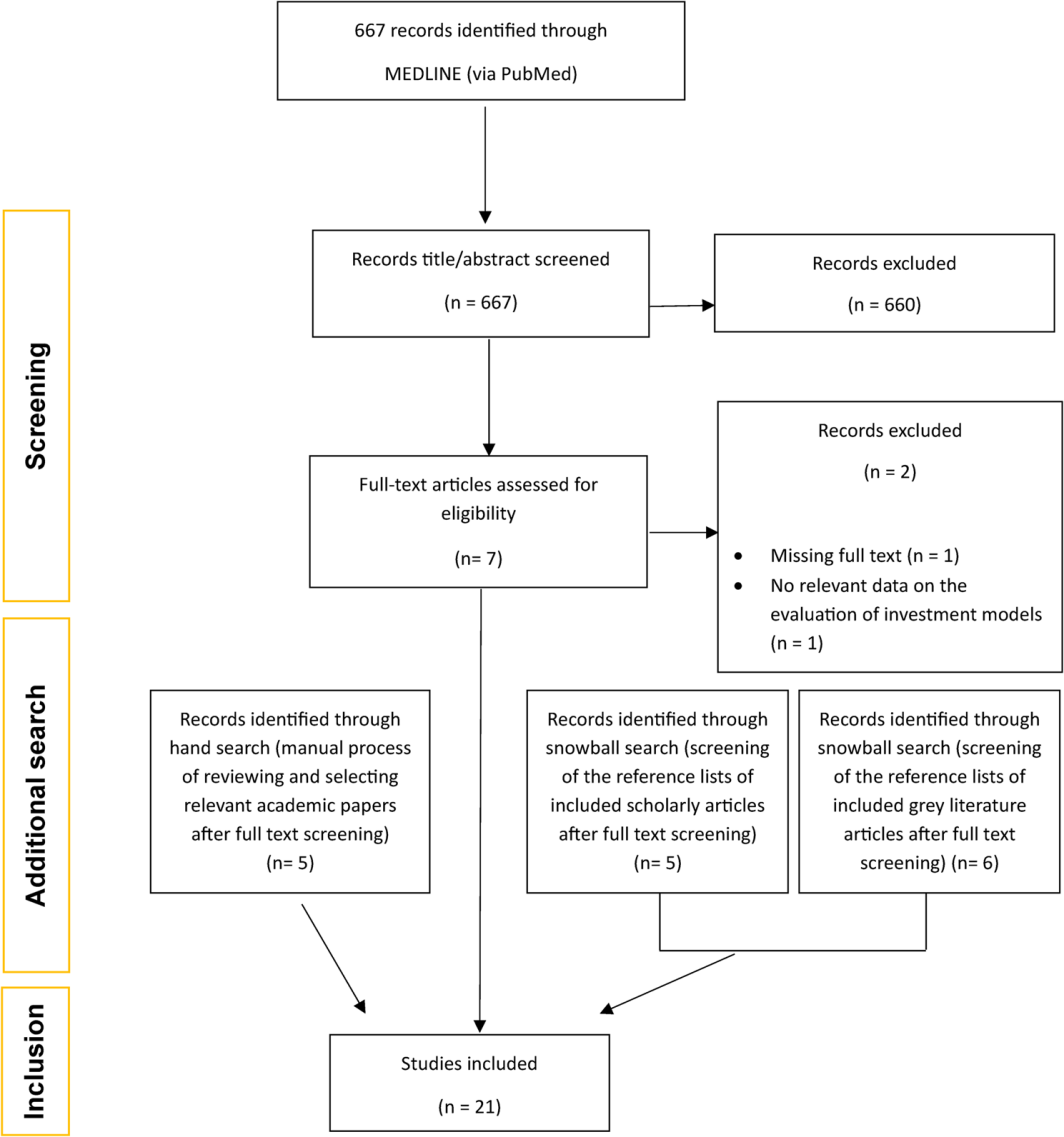
PRISMA flowchart

Figure 1 presents the results of the literature search. The PubMed search yielded 667 results, of which 7 were selected for full text screening after title/abstract screening. Five papers were included after full text screening. The snowball method resulted in 11 articles, 5 of them from scientific literature and 6 from grey literature. The hand search resulted in 5 more recent publications meeting the inclusion criteria. In total, therefore, the literature search yielded 21 results, shown in the PRISMA flowchart, all of which were used in the literature review.

Most of the papers discuss cases from the United Kingdom (UK), United States (US) and Australia, while a few of them have a global or another country specific perspective. Only 8 of the 21 included papers provided empirical results (Table 2).

**Table 2:** Characterization of the analyzed literature

ID	Author	Year of publication	Geographic focus	Type of article	Source
1	Fitzgerald	2013	Australia	conceptual/theoretical	PubMed
2	Galloway	2014	USA	conceptual/policy analysis	
3	Rowe and Stephenson	2016	mostly UK, Australia, and the USA but global	conceptual/theoretical	
4	Fischer and Richter	2016	USA	conceptual/theoretical	
5	Iovan and Lantz	2018	USA	letter to the editor	
6	Sugarman and Sandman	2008	Australia	conceptual/theoretical	Snowball
7	Bernet	2012	USA	conceptual/theoretical	
8	Azemati et al.	2013	USA	conceptual/policy analysis	
9	Nicholls and Tomkinson	2013	UK	empirical (case study)	
10	Golden	2014	USA	empirical (case study)	
11	Banke-Thomas et al.	2015	Global	conceptual/theoretical	
12	Gustaffson-Wright et al.	2015	Worldwide	conceptual/theoretical	
13	Lantz et al.	2016	USA	empirical (landscape analysis)	
14	Corporation for National and Community Service	2016	USA	empirical (systematic review, interviews and survey)	
15	Goldberg	2017	USA	conceptual/case-study	
16	Katz et al.	2018	global - UK, USA, Canada, Australia, EU and others	conceptual/theoretical	
17	Dowling	2017	UK	conceptual/theoretical	Hand search
18	Caré et al.	2020	UK, Australia	empirical (mixed-method grounded theory with survey	
19	Fraser et al.	2020	UK	empirical (case study)	
20	Fox et al.	2022	UK	empirical (case study)	
21	Economy et al.	2023	UK	empirical (qualitative content analysis)	

### Potential advantages of SIBs

#### Outcome-related aspects

By the nature of SIBs, their main conceptual advantage lies in potentially improving service outcomes. Outcomes may encompass a wide range of specific sector measures, including job conversion rate [28], recidivism rate [29], and increases in HIV diagnosis and treatment uptake among undiagnosed individuals [28] – some of which also relate to equity. Theories of change and corresponding logic models that SIBs are supposed to fit into are also varied. Below, we summarize the most important pathways to outcomes.

##### Flexibility in service provision

Compared with traditional global budgets or fee-for-service contracts, SIBs grant providers greater autonomy in determining how to achieve mutually agreed outcomes. Interviewees emphasized that this flexibility allows services to adapt to user needs, incorporate real-time data, and innovate iteratively (Interviewees 5, 6). Similar findings are also present in the literature, highlighting the potential for continuous learning, performance improvement and adaptation of service value networks involved in SIBs [21, 29–31].

##### Strengthening performance monitoring

Some studies claim that SIBs foster a culture of monitoring and accountability by embedding outcome-based contracts and intensive collaboration [19]. Service providers enhance data collection capacity and develop stronger evaluation practices [32]. Interviewee 3 confirmed that real-life UK experience, while sometimes reliant on outputs rather than outcomes, still catalysed the implementation of more rigorous monitoring frameworks.

##### Innovation in service delivery

SIBs may encourage providers to pursue innovative approaches, including co-creation and strengths-based models, and extend proven programmes to new populations or contexts [21, 30]. According to some scholars in the US, such as Albertson et al. [33], the type of innovation that can occur is when SIBs introduce elements of co-creation approaches to public service delivery [30].

##### Government capacity to test interventions

SIBs allow governments to pilot interventions or scale up existing programmes without assuming full financial risk [21, 29]. However, the piloting approach is most dominant in the United States, where data collection in SIBs is formally recognized as a tool for evidence building due to the use of randomized controlled trials alongside SIB pilots.

#### Financial aspects

While SIBs are designed mainly to improve outcomes, their impact on public finances is also a major area of scrutiny, given the scarcity of public health financing described in the Introduction. Although there are multiple potential mechanisms of improvement in this area, as summarized below, the extent of these advantages, especially of budgetary savings, is typically moderate.

##### Financial stability for providers

Interviewees highlighted that SIBs provided longer-term and more secure funding horizons, enabling small and third-sector providers to retain skilled staff and maintain services during austerity periods (Interviewees 1, 3). Literature similarly identifies enhanced stability as a key benefit [16, 30].

‘…*I think what I like about the model is first of all*,* it gives longer funding to a provider. So rather than one year funding and then we reapply*,* we were looking at three or five or even seven years funding. So it gives stability to the provider organizations*,* which I think is good*.” (Interview 1).

##### Potential government cost savings

Theoretical models suggest that early intervention through SIBs can deliver cashable long-term fiscal savings by reducing costly treatment later on [29]. However, both interviewees and empirical studies questioned the magnitude and realization of such savings. As Interviewee 3 emphasized, cashable savings often accrue unevenly, with national governments benefitting more than local commissioners.

’…*And it’s because of this assumption that the local commissioner is saving in the long term*,* but it’s not necessarily that that saving is in a bucket in a lot of the cases. […] in terms of cashable savings attributed to the lead commissioner*,* that’s around [amount]. And the savings for the national government is around [amount] cashable and then [amount] in non-cashable savings”* (Interview 3).

##### Risk transfer to private investors

A commonly cited advantage is shifting financial risk from governments to investors, who only receive repayment if outcomes are achieved [19]. This structure may incentivize governments to support innovative pilots during fiscal austerity. Nevertheless, national differences were noted: in the US, providers typically bore little or no risk, while in the UK some degree of provider risk-sharing was explicit [31].

#### System-level aspects

Beyond individual projects, SIBs may also have spillover effects and system-level implications. These may foster policy change, provided that a SIB manages to move beyond a trial state and scale up to have system-level implications.

##### Evidence-based policymaking

SIBs may reinforce the use of evidence in policymaking by directing funding to interventions with demonstrated cost-effectiveness and strengthening local data collection. Fraser et al. (2020) list two theoretical advantages related to evidence-based policymaking and compare them to actual practice [32]. The first is that SIBs support programmes that have already been tested and found to be successful. However, the reality is that SIBs are used to fund both proven and unproven interventions; the latter either because of the commissioner’s confidence or commitment to experimentation [32]. In addition, theory states that SIBs improve local data collection to promote evidence-based practice, which is supported by empirical evidence [28, 32].

##### Enhanced accountability

By requiring regular evaluation, SIBs may increase transparency and accountability of service providers [21]. According to Stoesz, a theoretical advantage is that the collection and use of more data increases the transparency and accountability of SIB-funded programmes [32, 34]. Introducing regulation into the measurement of outcomes and developing new data and performance benchmarks for social programmes may increase effectiveness [29]. However, issues were raised by both Interviewee1 and Interviewee4, who questioned the positive impact of more data when there is a lack of transparency about the cost-effectiveness of programmes.

##### Collaboration

Scholars suggest SIBs facilitate collaboration across government agencies, nonprofits, and private sector actors [19]. However, this was not explicitly highlighted by interviewees.

#### Potential disadvantages of SIBs

Most theoretical advantages of SIBs also have important limitations, in some cases even amounting to disadvantages. The same way as for advantages, we summarize these on project outcome, financial and system level.

#### Outcome-related aspects

##### Gaming and perverse incentives

Several authors warn that outcome-based payments may create perverse incentives for “cream skimming,” gaming, or focusing on easily measurable outcomes at the expense of broader impacts [32, 35–38]. These may hinder equity through an exclusion of vulnerable populations or an increase in territorial inequalities, as investors and often also commissioners are interested in demonstrating the effectiveness of the instrument through selecting target groups where the chances of success are higher [31]. Furthermore, gaming may include the service provider manipulating the results of the intervention or misreporting them [38]. Several studies highlight the potential of gaming [36, 37] and its negative effects, which may include weakening the validity of data [32], however, no empirical cases of gaming were identified.

##### Challenges in impact measurement

Some researchers argue that more learning from the service provision and better outcomes could be achieved if impact measurement is not limited to quantitative outcomes and more qualitative evidence is used in the evaluation of social impact bonds [30, 32]. They argue that over-emphasizing quantitative outcomes reduces the potential for learning and innovation and provides a good breeding ground for creaming [32]. However, according to Interviewee3, some of the distortion in impact measurement is mitigated by qualitative observations in the UK.

#### Financial aspects

##### High transaction costs

Designing and implementing SIBs is resource-intensive, involving complex contracting, intermediaries, evaluation, and legal support [19, 29, 39]. Interviewees questioned whether such costs outweighed the benefits, particularly when proven interventions could be publicly financed directly, according to Maier in Fraser’s study [32, 40].

‘’*And the question then becomes why do you need to ask the private sector to provide that upfront investment. […] if you knew that you were going to get the return then you don’t need to be paying private sector investors because you’ve achieved a saving in the public purse and all you need is a way of liberating money upfront which is what most of these schemes do in order to finance a saving which is later down the track*” (Interview 5).

##### Wrong-pocket problem

A recurrent issue is misaligned incentives, whereby one government body funds the intervention but savings accrue to another sector or level of government, complicating budgetary accountability [17]. SIBs often do not take into account increased costs in other areas and focus on specific areas of cost avoidance, leading to a misjudgment of the overall cost-effectiveness of an intervention [41]. Several interviewees highlighted this as a practical barrier to implementation.

#### System-level aspects

##### Political vulnerability

Political support is necessary for the establishment of SIBs, but an increased level of political attention, both at local and higher levels, may jeopardize the continuity of the intervention when there are changes in public administration [29]. Concerns range from governments’ biasing impact measurement if there is a lack of transparency on the cost-effectiveness of the programme, to the implementation or continuation of projects being subject to decisions by subsequent political administrations (Interviewee1; Interviewee4). However, the lack of transparency also means that cost-benefit analyses cannot be carried out properly due to a lack of data. Finally, from the interviews it seems that the implementation and continuation of SIB-funded interventions depend on the decisions of potentially-biased and changing political administrations. This may mean that high-level evaluation and decisions about the future of the instrument are highly political and that SIBs are not as cost-effective as hoped [29].

##### Scale-up difficulties

Expanding successful pilots remains challenging, as small providers often lack capacity for large-scale delivery. According to Interviewee2, there are two different alternatives: one is that the government takes over the management of the SIB-funded programme, and the other option is that the scale-up happens more organically and remains funded by social investors. In this case, however, scale-up depends on the richness of the ecosystem of social organizations, as Interviewee5 explains. One solution to the scale-up barrier is to promote approaches that aim to build capacity, which is seen as more feasible than relying solely on the original social enterprise to grow - as noted by Interviewee6. These strategies could include forming a larger consortium, involving more actors in service delivery, franchising the SIB model or training additional organizations. However, there is some evidence in the UK (a more-mature SIB ecosystem) that the SIB funding contributed to the improved scale-up of evidence-informed interventions in the National Health Service (NHS) in the UK (Hulse and Fraser 2024).

Table 3 below provides a summary of the advantages and disadvantages according to the three main aspects: outcome-related, financial system-level.

**Table 3:** Identified potential advantages and disadvantages of social impact bonds in health promotion and disease prevention

	Potential advantages	Potential disadvantages
Outcome-related aspects	Flexibility in service provision	Gaming and scheming
	Endorse performance monitoring	Difficulties with impact measuring
	Promoting innovation in service delivery	
	Governments can test with innovative interventions	
Financial aspects	Financial stability for service providers	Risk of high transactions cost
	Cost saving for the government	Wrong-pocket problem
	Transferring risks from government to private investors	
System-level aspects	Favor evidence-based policymaking	Highly politized sector
	Enhance accountability of providers	Scaleup difficulties
	Encourage collaboration	

Building on the above, we situate SIBs within a typology of investment adapted from [42]. Results are shown in Table 4.

**Table 4:** SIBs within the typology of investment models adapted from Rutten - van Mölken et al. [42]

Domains	Most relevant sub-domains	Conclusions regarding SIBs
Scope	The project’s target groups, maturity, and time frame	Projects with clearly defined and identifiable target groups and limited time frame (outcomes realized in 2–5 years) are best suitable for SIBs. Outcomes realised in the distant future, or influenced by many contextual factors, discourage private investors, while a very immediate connection between the intervention and its outcomes suggests that the intervention could also be performed by the public sector. In terms of project maturity, SIBs constitute a useful tool for experimentation, but interventions should already have at least a basic level of evidence base.
Partnership	Types of different partners involved, and their roles	The role of outcome evaluation is of particular importance here. While the involvement of an external evaluator usually comes at extra transaction costs, it contributes to the increase of outcomes focus throughout the system and also helps mitigate the risk of gaming.
Investment contract and outcomes	Size of the contract, level of return, degree to which the level of return is tied to the outcomes	Ideally, the return of SIBs is fully tied to the outcomes, either in a pass-or-fail or an incremental model, with special attention for excluding the opportunities for cream skimming and other types of gaming (e.g., clear rules for the inclusion of everyone in the target group into the service). Here again, there is a trade-off between a full reflection of actual outcomes (a theoretical complete contract) on the one hand, and transaction costs on the other.
Risk profile	Expected financial and outcomes risk	SIBs reach their objectives if substantial risk is transferred from the commissioner to the investor, which implies a strong link between outcomes and payments, and outcomes that are non-trivial to attain, otherwise, the benefits of the model do not manifest. On the other hand, an excessively high level of risk may discourage investors.
Feasibility	Model complexity and competences required	SIBs constitute a complex instrument that needs time and resources to develop. Therefore, only project owners with sufficient knowledge and resources and should embark on them.
Scalability	Transferability, repeatability and scalability	SIBs really realise their objectives if the interventions under experimentation can be transferred to other places or scaled to regional or national level afterwards.

#### Implications for policy and practice

- Social impact bonds are an innovative solution for fostering interaction and outcome-focused cooperation among different stakeholders, and can therefore create added value in prevention and health promotion.
- Although many potential drawbacks of SIBs can be found in the literature, empirical findings rather show an evolving and constantly developing instrument.
- While SIBs are not a system-level solution for the problem of underinvestment in prevention and health promotion, they offer good potential for better service value where outcomes and time frames can be relatively narrowly defined.

## Discussion

Outcomes-based financing is increasingly relevant as scarce healthcare resources have to be used efficiently. Barış et al. [3] stress the importance of prioritizing impactful health services within budget limitations, establishing accountability frameworks, and promoting transparency in public health financing. Within the Invest4Health project, we explored Smart Capacitating Investments for prevention and health promotion [42, 43]. Our findings suggest that SIBs may provide a flexible financing instrument to convene stakeholders, test innovation, and sustain long-term funding.

Still, evidence of SIB effectiveness in health prevention remains scarce. Hulse et al. [44] found little empirical support for SIBs in addressing NCDs, citing conflicts of interest and limited transparency. Gustafsson-Wright and Osborne [45] questioned whether SIBs can address major public health problems such as obesity, while Ronicle et al. [46] observed that SIBs are better suited to narrowly-focused initiatives than to population-wide prevention. Challenges include defining target groups, measuring diffuse long-term outcomes, and attributing savings. Accordingly, SIBs may be most appropriate for tertiary prevention or targeted primary prevention programmes with measurable intermediate outcomes.

Expected cost savings, often central to initial SIB justification, rarely materialize. While models predict efficiency gains from early intervention [29], both interviews and prior studies show that budget savings are limited or realized unevenly across levels of government [17]. High transaction costs further limit efficiency, as SIB design and implementation require substantial legal, evaluative, and contractual resources [19, 20].

Scaling up successful SIB programmes is another challenge. Regional conditions—such as availability of private philanthropic or near-commercial capital, robust civil society, and political support—shape feasibility, explaining why most SIBs originate in the UK and US. Even where ecosystems are favourable, political turnover can jeopardize continuity [29].

While the above substantial challenges exist, the SIB ecosystem is also a dynamically evolving space, with more recent empirical findings pointing towards increasingly careful preparation, managing risks and enhancing system-level benefits such as evidence-based policymaking and accountability risk. This phenomenon of time inconsistency may explain why interviewees, who are closer to the latest developments of the sector, tended to be – although not always – more optimistic about SIBs than what can be found in the literature, which, due to publication time lags, often addresses the challenges of earlier SIB examples. Some interviewees working directly on the creation of SIBs may also be positively biased towards them; however, we believe that the inclusion of critical researchers in our sample effectively mitigated this bias.

Outcome funds may mitigate multiple barriers by pooling projects, spreading risk, and attracting larger investors. Globally, 21 outcome funds exist, with new initiatives in the UK and Australia, including the AUD 100 million Commonwealth Outcomes Fund [47, 48].

A limitation of this study is the scarcity of literature on SIBs for preventative healthcare, which weakens the robustness of our conclusions. Furthermore, our literature review has methodological limitations that might affect its comprehensiveness, including:

- Only one major database (PubMed) was searched.
- Screening was not double-reviewed.
- No formal quality appraisal of included studies was conducted.
- Inclusion and exclusion criteria, while described, are not operationalized in a way that enables replication.
- Study selection flow is summarized narratively but not structured in a PRISMA-style format.

The small number of interviews within the Invest4Health project also limits generalizability, though their geographical diversity is a strength. Triangulation of published, grey, and expert evidence helps mitigate these limitations.

Further research is needed to provide empirical evidence about the financial and social outcomes of individual SIBs, as well as their system-level implications once scaled up to regional or national level. This could support a better understanding of where and in what cases the theoretical critiques of the literature still hold merit in practice, and what are the cases where development and fine-tuning could effectively address them. 

## Conclusion

SIBs represent an investment and finance model that is still new in the area of health promotion and disease prevention. According to our findings, they may offer flexibility and innovation in service provision, which is much needed in solving complex problems. However, SIBs also face potential challenges, for instance high transaction costs, uncertain financial savings, political vulnerability, and limited scalability. They are not sufficient in themselves for resolving the problem of under-investment in public health, and their objective is not to negate the need of public resource reallocation. Another problem is that public health interventions, especially in primary prevention focusing on the general population, often have effects in the distant future, and they are also difficult to attribute to a single and measurable cause. However, in cases where outcomes can be defined in a narrower sense, e.g., primary prevention focusing on at-risk sub-populations, or tertiary prevention, our conclusion is that they may bring additional added value through flexibility, transparency, outcome focus and multi-stakeholder cooperation.

## Supplementary Information

Supplementary Material 1: Appendix A. Informed consent form for the interviews.

Supplementary Material 2: Appendix B. Interview guide.

Supplementary Material 3: Appendix C. Coding matrix.

Supplementary Material 4: Appendix D. Search terms.

## Data Availability

All available data is contained in the supplementary material.

## References

[1] Adrian T, Gaspar V, Gourinchas P-O. The Fiscal and Financial Risks of a High-Debt, Slow-Growth World. 2024.

[2] Federal Reserve Bank of St L. 10-Year Treasury Constant Maturity Rate (DGS10). 2025.

[3] Baris E, Silverman R, Wang H, Zhao F, Pate MA. Walking the Talk: Reimagining Primary Health Care After COVID-19. Washington, DC: World Bank; 2021.

[4] OECD. Health at a Glance: Europe 2022. Paris: OECD Publishing; 2022.

[5] Ogden CL, Carroll MD, Kit BK, Flegal KM. Prevalence of childhood and adult obesity in the United States, 2011–2012. JAMA. 2014;311(8):806–14. 24570244 10.1001/jama.2014.732 PMC4770258

[6] Petrelli NJ, Winer EP, Brahmer J, Dubey S, Smith S, Thomas C, et al. Clinical Cancer Advances 2009: major research advances in cancer treatment, prevention, and screening–a report from the American Society of Clinical Oncology. J Clin Oncol. 2009;27(35):6052–69. 19901123 10.1200/JCO.2009.26.6171

[7] Thornton RL, Glover CM, Cene CW, Glik DC, Henderson JA, Williams DR. Evaluating Strategies For Reducing Health Disparities By Addressing The Social Determinants Of Health. Health Aff (Millwood). 2016;35(8):1416–23. 27503966 10.1377/hlthaff.2015.1357 PMC5524193

[8] Wang F, Wang JD. Investing preventive care and economic development in ageing societies: empirical evidences from OECD countries. Health Econ Rev. 2021;11(1):18. 34086126 10.1186/s13561-021-00321-3 PMC8176873

[9] EUROSTAT. Expenditure for selected health care functions by health care financing schemes Official website of Eurostat2025 [Available from: https://ec.europa.eu/eurostat/databrowser/view/hlth_sha11_hchf__custom_19710136/default/table

[10] OECD. Fiscal Sustainability of Health Systems: How to Finance More Resilient Health Systems When Money is Tight? OECD Publishing; 2021.

[11] Borins S. New public management: North American style. In: McLaughlin K, Osborne SP, Ferlie E, editors. The new public management: Current trends and future prospects. London: Routledge; 2000. pp. 181–94.

[12] Simonet D. The New Public Management Theory in the British Health Care System. Adm Soc. 2013;47(7):802–26.

[13] Child C, Gibbs BG, Rowley KJ. Paying for success: An appraisal of social impact bonds. Global Econ Manage Rev. 2016;21(1–2):36–45.

[14] GoLab. The evolution of social outcomes partnerships in the UK. 2024.

[15] Floyd D, Social Impact, Bonds. An Overview of the Global Market for Commissioners and Policymakers 2017 [Available from: https://socialspider.com/wp-content/uploads/2017/04/SS_SocialImpactReport_4.0.pdf

[16] Fitzgerald JL. Social Impact Bonds and Their Application to Preventive Health. Aust Health Rev. 2013;37(2):199–204. 23575507 10.1071/AH12238

[17] Lantz PM, Rosenbaum S, Ku L, Iovan S. Pay For Success And Population Health: Early Results From Eleven Projects Reveal Challenges And Promise. Health Aff (Millwood). 2016;35(11):2053–61. 27834246 10.1377/hlthaff.2016.0713

[18] Deckers L, Motivation. Biological, Psychological, and Environmental. 4 ed. London; New York: Routledge; 2016.

[19] Gustafsson-Wright E, Gardiner S, Putcha V. The Potential and Limitations of Impact Bonds: Lessons from the First Five Years of Experience Worldwide. Brookings Institution; 2015.

[20] Goldberg SH. Scale Finance: Industrial-Strength Social Impact Bonds for Mainstream Investors2017.

[21] Katz AS, Brisbois B, Zerger S, Hwang SW. Social Impact Bonds as a Funding Method for Health and Social Programs: Potential Areas of Concern. Am J Public Health. 2018;108(2):210–5. 29267055 10.2105/AJPH.2017.304157 PMC5846579

[22] Palinkas LA, Horwitz SM, Green CA, Wisdom JP, Duan N, Hoagwood K. Purposeful Sampling for Qualitative Data Collection and Analysis in Mixed Method Implementation Research. Adm Policy Ment Health. 2015;42(5):533–44. 24193818 10.1007/s10488-013-0528-y PMC4012002

[23] Github.com. Buzz Transcribes and Translates Audio Offline on Your Personal Computer. Powered by OpenAI’s Whisper; 2024.

[24] Bradley EH, Curry LA, Devers KJ. Qualitative data analysis for health services research: developing taxonomy, themes, and theory. Health Serv Res. 2007;42(4):1758–72. 17286625 10.1111/j.1475-6773.2006.00684.x PMC1955280

[25] Saldana J. The Coding Manual for Qualitative Researchers. 3 ed. London: Sage; 2016.

[26] OECD. Social Impact Bonds. State of Play & Lessons Learnt. OECD; 2016.

[27] Wohlin C, Kalinowski M, Felizardo KR, Mendes E. Successful Combination of Database Search and Snowballing for Identification of Primary Studies in Systematic Literature Studies. ‎Inf Softw Technol. 2022;147:106908.

[28] Hulse E, Fraser A. How Can We Scale Up Evidence-Informed Health Care Interventions Through Social Outcomes Partnerships? In: Anastasiu A, Carter E, Airoldi M, editors. The Evolution of Social Outcomes Partnerships in the UK. Oxford: Government Outcomes Lab, Blavatnik School of Government, University of Oxford; 2024. pp. 62–4.

[29] Nicholls A, Tomkinson E. The Peterborough Pilot Social Impact Bond. In: Nicholls A, Paton R, Emerson J, editors. Social Finance. Oxford: Oxford University Press; 2015. pp. 335–80.

[30] Fox C, Olson H, Armitage H, Baines S, Painter G. Can a focus on co-created, strengths-based services facilitate early-stage innovation within social impact bonds? Int Public Manage J. 2022;26(3):396–412.

[31] Economy C, Carter E, Airoldi M. Have we ‘stretched’ social impact bonds too far? An empirical analysis of SIB design in practice. Int Public Manage J. 2022;26(3):413–36.

[32] Fraser A, Tan S, Boaz A, Mays N. Backing what works? Social Impact Bonds and evidence-informed policy and practice. Public Money Manage. 2020;40(3):195–204.

[33] Albertson K, Fox C, O’Leary C, Painter G, Bailey K, LaBarbera J. Payment by results and social impact bonds. University of Chicago Press Economics Books; 2018.

[34] Stoesz D. Evidence-Based Policy:Reorganizing Social Services Through Accountable Care Organizations and Social Impact Bonds. Res Social Work Pract. 2014;24(2):181–5.

[35] Dowling E. the wake of austerity: social impact bonds and the financialisation of the welfare state in Britain. New Polit Econ. 2016;22(3):294–310.

[36] Sandman N, Sugarman SD. Using Performance-Based Regulation to Reduce Childhood Obesity. Australia New Z Health Policy. 2008;5(1). 10.1186/1743-8462-5-26 PMC2596772 19017402

[37] Service CfNaC. State of the Pay For Success Field II: Emerging Literature, Updates, and Tools. Washington, DC: Office of Research and Evaluation; 2016.

[38] Carè R, Rania F, De Lisa R. Critical Success Factors, Motivations, and Risks in Social Impact Bonds. Sustainability. 2020;12(18):7291.

[39] Azemati H, Belinsky M, Gillette R, Liebman JB, Sellman A, Wyse A. Social Impact Bonds: Lessons Learned So Far. Community Dev Innov Rev. 2013;1:23–33.

[40] Maier F, Barbetta GP, Godina F. Paradoxes of Social Impact Bonds. Social Policy Adm. 2017;52(7):1332–53.

[41] Fischer RL, Richter FG. SROI in the pay for success context: Are they at odds? Eval Program Plann. 2017;64:105–9. 27899208 10.1016/j.evalprogplan.2016.11.012

[42] Rutten-van Molken M, Whiteley H, Babarczy B, Davies J, Goossens L, Papartyte L et al. Broadening sources of finance for health promotion and disease prevention: Smart capacitating investment. Eur J Health Econ. 2025. 10.1007/s10198-025-01874-4 PMC13451443 41460270

[43] Lane J, Edwards RT, Babarczy B, Whiteley H, Oruganti V, Rutten-van Molken M, et al. A protocol for mobilising novel finance models for collaborative health promotion and disease prevention initiatives: taking a smart capacitating investment approach in the Invest4Health project. Front Public Health. 2024;12:1426863. 39917535 10.3389/fpubh.2024.1426863 PMC11799243

[44] Hulse ESG, Atun R, McPake B, Lee JT. Use of social impact bonds in financing health systems responses to non-communicable diseases: scoping review. BMJ Glob Health. 2021;6(3):e004127. 33674267 10.1136/bmjgh-2020-004127 PMC7938989

[45] Gustafsson-Wright E, Osborne S. Are impact bonds reaching the intended populations? Brookings.edu: Brookings; 2020 [Available from: https://www.brookings.edu/articles/are-impact-bonds-reaching-the-intended-population/

[46] Ronicle J, Stanworth N, Wooldridge R. Commissioning Better Outcomes Evaluation. 2022.

[47] Savell L, Carter E, Airoldi M, FitzGerald C, Tan S, Velarde JO et al. Understanding Outcomes Funds: A Guide for Practitioners, Governments and Donors. 2022.

[48] Australian Government DoSS. Commonwealth Outcomes Fund. 2025.

