# Supplementary material for: Social impact bonds: opportunities for funding health promotion and disease prevention
Source: BMC Public Health. 2026 Mar 16;26:1329. doi: 10.1186/s12889-026-26916-1 (PMC13104328; doi:10.1186/s12889-026-26916-1)
Supplement: Supplementary file 1 — Supplementary Material 1: Appendix A. Informed consent form for the interviews. [file 12889_2026_26916_MOESM1_ESM.docx]

# Social Impact Bonds: Opportunities for funding health promotion and disease prevention

# Appendix A – Informed consent form for the interviews


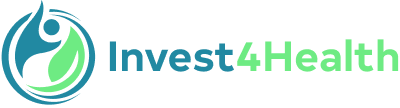


**Informed Consent Form for Expert Interviews on Finance and Investment Models**

**Purpose of our research**

Invest4Health is a collaborative research project funded by the European Union (Horizon Europe no. 101095522). The aim of the project is to develop and innovative investment models which could help boost prevention and health promotion interventions in Europe, channeling new funds into the improvement of the health and wellbeing of populations. We would like to have a better understanding of the most promising investment structures globally, and on these grounds develop financing and investment models for the project’s regional test-beds.

**Type of study**

To complement the findings of a scoping review of the literature on smart capacitating investment, Syreon Research Institute is conducting semi-structured interviews with experts of impact investment and health financing mechanisms.

**Procedures**

A semi-structured interview is a slightly directed conversation, which will last around 60 minutes. The interview, if you agree to this, will be audio recorded, and will then be transcribed and analyzed. The recording will be deleted immediately after the transcript has been made.

**Participant selection**

You have been identified as a potential participant of this research based on your expertise and/or research activities in the fields of impact-oriented investment and/or health financing systems.

**Voluntary participation**

Participation is entirely voluntary. You may also stop participating in the interview any time you wish.

**Reimbursements**

You will not be provided any monetary incentive to take part in the research. We will do our best to respect the value of your time and not to spend more of it than agreed.

**Confidentiality**

Participation in this research is confidential. Any information you share will be confidential and cannot be traced back to you when results and findings are summarized. The transcript of this interview will be anonymized, and stored at a secure drive of Syreon Research Institute. It will only be accessible to members of the research working group, i.e., employees of Syreon Research Institute and, eventually, some partner organizations of Invest4Health that they give access to, for the strict purposes of the research study. Records and transcripts will be deleted by the end of the project (30 June 2026) latest.

**Data protection**

Data is collected with reference to and in respect of GDPR Article 9(2)(j). No information from this interview will be shared outside the research team, and nothing will be attributed to you by name. The knowledge that we get from this research will be summarized in a final report, and potential scientific publications about the study results. You may indicate, during or after the interview, and until completion of the research study, if there are parts of the information that you do not want to be used. You also have a right to have any personal data about you corrected or deleted, unless this request would make it impossible or make it very difficult to conduct the research.

**Risks**

We have not identified any risks associated with taking part in this study. However, if you may feel uncomfortable talking about some of the topics, you do not have to answer any question or take part in the interview if you do not wish to do so. You do not have to give any reason for not responding to any question, or for refusing to take part in the interview.

**Contact**

**Project website:** https://invest4health.eu/.

**Principal investigator of the present interview study**: Balázs Babarczy, senior researcher, Syreon Research Institute (Budapest), [balazs.babarczy@syreon.eu](mailto:balazs.babarczy@syreon.eu)

**Project coordinator**: Jolanda van Vilet, healthcare strategist, Region Skåne (Sweden), [jolanda.vanvliet@skane.se](mailto:jolanda.vanvliet@skane.se)

**Informed consent**

**I have been invited to participate in a qualitative interview study with the aim to explore social impact-oriented investment models and their applicability in public health. I have read the foregoing information.**

**I consent to the recording of this conversation.**

**I consent to the use of the information I provide, for the purposes of the research study, under the conditions of confidentiality detailed above.**

**Print Name of Participant**__________________

**Signature of Participant __________________ _**

**Date ___________________________**

**Day/month/year**
