# Supplementary material for: Social impact bonds: opportunities for funding health promotion and disease prevention
Source: BMC Public Health. 2026 Mar 16;26:1329. doi: 10.1186/s12889-026-26916-1 (PMC13104328; doi:10.1186/s12889-026-26916-1)
Supplement: Supplementary file 2 — Supplementary Material 2: Appendix B. Interview guide. [file 12889_2026_26916_MOESM2_ESM.docx]

# Social Impact Bonds: Opportunities for funding health promotion and disease prevention

# Appendix B – Interview guide of qualitative expert interviews

*Engagement*

- **Do you have experience with** or conducted research about **investment models with explicit social and/or health impact?**
- ***if yes:* Please describe your experience with/research findings** about investment models having social and/or health impact.
  - What was your institution’s role in these projects?
  - What was your role in these projects?
  - How would you evaluate the success of these projects?
- ***if no:*** Did you manage to read the study sent before this interview? **What did you find most interesting about these models?**

*Exploration*

- Please think about one or more healthcare-related or social investment projects that you have been recently involved in or researched on. **Please describe the intervention.**
  - What were the **main goals** of the intervention (health or non-health related)?
  - What was the **target population**?
  - Who were the **main stakeholders**?
  - What was the **benefit period** of the project?
- **Please describe the investment model** applied.
  - Who were the **investors?**
    - Potential types include, but are not limited to: Government; Non-health public sector; Health insurer; Commercial bank; Social bank; Social enterprise; Social impact investor; Venture capital; Venture philanthropist; Accelerator; Third sector; Cooperative / mutual; Foundation; Voluntary / community group; Crowd funding; Charity
  - What were the **assets invested?**
    - e.g., money, know-how, employee time
  - What was the **amount** of resources invested?
  - Was it a **one-time or a recurring** investment?
  - **Did the investor play any other role in the project** (e.g., commissioner, managing/intermediary organisation, service provider)?
  - What was the **investment model?**
    - e.g., social impact bonds, social outcomes contracting, ESG – environmental, social, governance - type investment
  - What was the **timescale** of the investment model?
  - How much was the **return on investment** (in total or per year)?
  - What was the **basis of the returns** (financial savings during the service delivery or specified health/social outcomes reached)?
  - What **other benefits** did the investor realise (e.g., improved public image, gain of local market knowledge, gain of experience in the service area)?
- Please describe the **payment model applied within the project** *(if applicable)****.***
  - Who was the **commissioner** and who were the **service providers**?
  - What was the **basis of payment** between the commissioner and the service provider?
    - e.g., amount of services provided, number of people reached by the intervention, specific outcomes reached in the target population
  - What was the **valuation method** for such indicators?
  - **How much money was involved** in paying for the services (in total or per year)?
  - Did the model channel **extra funds** towards the preventive service, relative to baseline spending before its introduction?
  - **Was the model financially viable** (profitable/sustainable) **to the service provider?** And to the commissioner?
- Please describe **to what extent the investment model was successful.**
  - **Were health/social outcomes defined in advance?**
  - To what extent were these reached?
  - **How were these measured, and by whom?** Was there a standardized measurement framework/evaluation criteria used?
  - To what extent **were the investors satisfied** with the project?
  - To what extent **were other stakeholders (e.g., service providers, commissioner) satisfied** with the project?
  - How high were the **transaction costs** of the project?
  - What was the **public/political echo** of the project, if any?
  - What were the main **drivers of the project implementation**?
    - e.g., rate of return, security of the investment, transparency of the project, legal framework, personal leadership
  - What were the main **barriers to project implementation?**
    - e.g., legal, political, financial obstacles
  - In what areas (geographic, sector-specific, stakeholder-specific, etc.) **would you recommend this model to be implemented again?**
  - What do you think about the future potential of such projects? How would you describe the **market outlook** of investment in similar projects?
- In general, **what role do you think private investment could play in healthcare and public health?**
- **What criteria** **investment models should fulfill** in order to be successful in health?
- **What advantages could these models have,** relative to the traditional models of financing healthcare and public health?
  - e.g. arrival of new funds, know-how of the private investor channeled into the model, increased transparency and accountability of actors)
- **What should be done for the outcome/impact of these investment models to be better measured?**
  - Who should measure them?
- How could **transaction costs of these models be minimized?**
- **What other interventions would be necessary for this market to develop?**
  - potential actions: regulation, steering, definitions and labelling, capacity building, creation of new investment funds, networking and market development, etc.)
  - Should these be implemented by the state/EU, or by market actors themselves?

*Closing*

- Thank you very much for your time and for answering our questions!
- **Do you have any questions** yourself?
- **Could you recommend experts** who we should also interview on these topics?
