# Supplementary material for: Social impact bonds: opportunities for funding health promotion and disease prevention
Source: BMC Public Health. 2026 Mar 16;26:1329. doi: 10.1186/s12889-026-26916-1 (PMC13104328; doi:10.1186/s12889-026-26916-1)
Supplement: Supplementary file 3 — Supplementary Material 3: Appendix C. Coding matrix. [file 12889_2026_26916_MOESM3_ESM.docx]

# Social Impact Bonds: Opportunities for funding health promotion and disease prevention

# Appendix C – Coding matrix

This matrix was used for deductively coding the transcripts of expert interviews (step 2 or the qualitative data analysis process). Columns show consensus themes, while rows present different finance mechanisms mentioned by interviewees. (The present article only focuses on SIBs among these.)

| **Instruments** | | **A) Stakeholders** | **B) Motivation for investor / Investment case / Investment incentives** | **Context** | | | | | **D) Business model** | | **E) Investing model / Investment structure/technique** | | **F) Investment size** | | **G) Timeframe** | | **H) ROI / Returns** | | **I) Impact measurement** | | **J) Scaleup** | | **K) Challanges / Critique** | | | **L) Other** | | **Innovation maturity (early stage business or mature)** |
| --- | --- | --- | --- | --- | --- | --- | --- | --- | --- | --- | --- | --- | --- | --- | --- | --- | --- | --- | --- | --- | --- | --- | --- | --- | --- | --- | --- | --- |
|  |  |  |  | Investment ecosystem | Regulatory issues | Geographical spread | Wrong pocket problem |  | |  | |  | |  | |  | |  | |  | | Advantages | | Disatvantages |  | |  | |
| Funding with no return | 1. public money: treasury bonds / state bonds / low interest long term internal financing |  |  |  |  |  |  |  | |  | |  | |  | |  | |  | |  | |  | |  |  | |  | |
|  | 2. philanthropic investment (no return expectation e.g. grants) |  |  |  |  |  |  |  | |  | |  | |  | |  | |  | |  | |  | |  |  | |  | |
| Funding with preferential return | 3. SIBs (including bundle) |  |  |  |  |  |  |  | |  | |  | |  | |  | |  | |  | |  | |  |  | |  | |
|  | 4. outcome based contracting (SOCs, PbR) |  |  |  |  |  |  |  | |  | |  | |  | |  | |  | |  | |  | |  |  | |  | |
|  | 5. social banking (subordinate, long term loans) |  |  |  |  |  |  |  | |  | |  | |  | |  | |  | |  | |  | |  |  | |  | |
|  | 6. social financing (small-scale social venture capital) |  |  |  |  |  |  |  | |  | |  | |  | |  | |  | |  | |  | |  |  | |  | |
|  | 7. impact investment (larger scale, more mature projects, ez is VC) |  |  |  |  |  |  |  | |  | |  | |  | |  | |  | |  | |  | |  |  | |  | |
| Funding with non-preferential return | 8. traditional market-based investment (no social impact required) |  |  |  |  |  |  |  | |  | |  | |  | |  | |  | |  | |  | |  |  | |  | |
| Other models | 9. microfinance |  |  |  |  |  |  |  | |  | |  | |  | |  | |  | |  | |  | |  |  | |  | |
|  | 10. community investment notes (offered by CDFI) |  |  |  |  |  |  |  | |  | |  | |  | |  | |  | |  | |  | |  |  | |  | |
|  | 11. crowdfunding for impact |  |  |  |  |  |  |  | |  | |  | |  | |  | |  | |  | |  | |  |  | |  | |
|  | 12. multistakeholder models (Stakeholder/Shareholder, Triple helix model, etc.) |  |  |  |  |  |  |  | |  | |  | |  | |  | |  | |  | |  | |  |  | |  | |
| Basically Non-financial investment | 13. incubation, acceleration activities, support (sweat equity) |  |  |  |  |  |  |  | |  | |  | |  | |  | |  | |  | |  | |  |  | |  | |
