# Supplementary material for: Social impact bonds: opportunities for funding health promotion and disease prevention
Source: BMC Public Health. 2026 Mar 16;26:1329. doi: 10.1186/s12889-026-26916-1 (PMC13104328; doi:10.1186/s12889-026-26916-1)
Supplement: Supplementary file 4 — Supplementary Material 4: Appendix D. Search terms. [file 12889_2026_26916_MOESM4_ESM.docx]

# Social Impact Bonds: Opportunities for funding health promotion and disease prevention

# Appendix D – Search terms

The search string below was applied for searching the literature in PubMed (MEDLINE).

(((((((("healthcare impact bond*" AND (english[Filter]))) OR ("payment by result" AND (english[Filter]))) OR ("payment by result*" AND "contract*")) OR ("payment by result*")) OR ("social outcome*" AND "contract*")) OR ("outcome based financ*" AND (e - Search Results - PubMed (nih.gov)
